# Resource consumption and management associated with monitoring of warfarin treatment in primary health care in Sweden

**DOI:** 10.1186/1471-2296-7-67

**Published:** 2006-11-12

**Authors:** Stina Andersson, Ingela Björholt, Gunnar H Nilsson, Ingvar Krakau

**Affiliations:** 1 AstraZeneca Sverige AB, Södertälje, Sweden; 2 Institute of Clinical Sciences, Göteborg University, Göteborg, Sweden and Nordic Health Economic Research AB, Göteborg, Sweden; 3 Neurotec Department, Karolinska Institutet, Stockholm, Sweden; 4 Center of Family Medicine, Karolinska Institutet, Huddinge and Department of Medicine, Solna, Sweden

## Abstract

**Background:**

Warfarin is used for the prevention and treatment of various thromboembolic complications. It is an efficacious anticoagulant, but it has a narrow therapeutic range, and regular monitoring is required to ensure therapeutic efficacy and at the same time avoid life-threatening adverse events. The objective was to assess management and resource consumption associated with patient monitoring episodes during warfarin treatment in primary health care in Sweden.

**Methods:**

Delphi technique was used to systematically explore attitudes, demands and priorities, and to collect informed judgements related to monitoring of warfarin treatment. Two separate Delphi-panels were performed in three and two rounds, respectively, one concerning tests taken in primary health care centres, involving 34 GPs and 10 registered nurses, and one concerning tests taken in patients' homes, involving 49 district nurses.

**Results:**

In the primary health care panel 10 of the 34 GPs regularly collaborated with a registered nurse. Average time for one monitoring episode was estimated to 10.1 minutes for a GP and 21.4 minutes for a nurse, when a nurse assisted a doctor. The average time for monitoring was 17.6 minutes for a GP when not assisted by a nurse. Considering all the monitoring episodes, 11.6% of patient blood samples were taken in the individual patient's home. Average time for such a monitoring episode was estimated to 88.2 minutes. Of all the visits, 8.2% were performed in vain and took on average 44.6 minutes. In both studies, approximately 20 different elements of work concerning management of patients during warfarin treatment were identified.

**Conclusion:**

Monitoring of patients during treatment with warfarin in primary health care in Sweden involves many elements of work, and demands large resources, especially when tests are taken in the patient's home.

## Background

The prevalence of patients on anticoagulant (AC) treatment in Sweden has been estimated to be between 0.75 and 0.88% (age adjusted) in a primary health care (PHC) setting [1,2]. Oral ACs, i.e. vitamin K-antagonists, are used for the prevention and treatment of various thromboembolic complications. Their efficacy has been demonstrated in a broad range of indications. These include the prevention of venous thromboembolism including pulmonary embolism, thrombosis on heart valve prostheses, and the prevention of stroke in chronic atrial fibrillation (CAF) [3-5]. Furthermore, vitamin K-antagonists have been demonstrated to be effective as secondary prophylaxis after myocardial infarction [6].

The standard oral AC in Sweden is warfarin. It is an efficacious anticoagulant, but it has a narrow therapeutic range, and interacts with a number of common drugs as well as with food and alcohol. If under-coagulated the patient is at risk of a thromboembolic event, while if over-coagulated there is a risk of bleeding complications. Therefore, patients on such treatment require regular monitoring of International Normalized Ratio (INR) values, in order to ensure therapeutic efficacy and at the same time avoid life-threatening adverse events. In Sweden such monitoring episodes are either managed by hospital anticoagulation clinics, in PHC, or in the patient's home.

The frequency of INR monitoring visits has been shown to be considerable for patients already established on warfarin treatment and managed in PHC in Sweden [1]. The burden on the health care sector to manage these patients is sizeable, as the number of patients requiring such monitoring episodes is large and each patient demands frequent monitoring. Furthermore, each monitoring episode is likely to be resource consuming, as a number of various elements of work are involved and meticulous management is critical for safety reasons. Health care resources are scarce and therefore elements of work for various aspects of health care should be continuously scrutinized. Thus a basis can be created for decisions on the allocation of health care resources to ensure that they are efficiently used. To our knowledge the association of resource consumption with INR monitoring episodes of patients during warfarin treatment in PHC has not previously been studied.

The objective of this study was to assess the management and resource consumption that is associated with INR monitoring episodes in patients during warfarin treatment, in PHC, in Sweden.

## Methods

The Delphi technique is a well-known method to systematically explore attitudes, demands and priorities of groups of experts [7-11]. The experts are usually selected to reflect current knowledge and perceptions in the field under consideration. The Delphi technique is based on a series of stages or iterations, where informed judgements on specific issues are collected from experts. The experts respond individually and anonymously to questions, to avoid influence of contextual factors such as personal characteristics, seniority and experience. Thereby, honest opinion free from peer group pressure is encouraged. The answers from all respondents in round one are put together and the respondents given feedback. In the following round the individual respondent has the opportunity to change his/her response. The depth of knowledge therefore increases among the respondents. Delphi-panels have commonly been used to build and measure consensus. However, the design may vary with the objective of the study, and one option is to canvass the relative importance or desirability of specific items by rank or by attitude statements according to ordinal scales, such as the Likert Scale [12,13].

Two separate Delphi-panels were performed. One concerned tests taken at a PHC centre (panel I), and the other concerned tests taken in the patient's home (panel II). Panel I included members randomly selected from general practitioners (GPs) and nurses in PHC in Stockholm County Council, and panel II included district nurses throughout Sweden. Navigare Medical Marketing Research AB, a medical research company, performed all contacts and interviews with respondents. A project group was nominated to develop the questionnaires and to assess the answers from each round. This group comprised one representative from the medical research company, one from AstraZeneca Sverige and two from the Center for Family Medicine, Karolinska Institutet, Stockholm. The identities of the panel members were not disclosed to the members of the project group.

The panel members were asked to i) identify elements of work associated with INR monitoring; indicate (ii) the time required to carry out each individual element of work; and (iii) the frequency by which they normally occur in a standard INR monitoring episode.

Information from previous rounds was fed back to the individual respondents to encourage a thorough reassessment of the individual answer. Hence the respondents were repeatedly prompted to assess whether the time they had indicated for the individual work processes added up to a reasonable estimate of time.

The frequency with which individual items of the work processes normally occurred was measured by a Likert Scale, where 0 is "never" and 10 is "always".

### INR tests taken at a PHC centre (panel I)

The objective of panel I was to assess resource consumption associated with INR monitoring episodes in patients during warfarin treatment when the INR test was taken at a PHC centre.

A random sample of 50 GPs from all PHC centres in Stockholm County was invited to participate in the study. Out of a total of 940 GPs and 174 PHC centres three GPs were selected at each of the 50 PHC centres, one as the primary respondent, and two additional as stand-in if the primary respondent should be impossible to reach or decline to participate. Where the GP routinely worked with a registered nurse in managing warfarin patients this nurse was invited to also participate in the study. The criterion for inclusion was management of five or more patients on warfarin treatment.

This Delphi-panel was performed in three rounds. The first and second rounds were accomplished by telephone interviews and the third round by a postal questionnaire. The GPs participated in all three rounds while the nurses only participated in the first and second round. In each of the three rounds, the interviewer tried up to three times to get in contact with the primary respondent by telephone. If no contact was established, a reminder letter was sent to the respondent. If contact still failed, the stand-in GP was asked to participate. The PHC centre panel first round was carried out in June 2003, the second round in September, and the third and last round was in December 2003.

In the first round, the GP respondents were asked to identify various elements of work that were involved in INR monitoring using an open-ended questionnaire. In each element of work several sub-elements could be included. For example, a GP could state when preparing for INR monitoring that he both read up on the patient and went through test results from a prior monitoring visit. It was therefore possible for respondents to give more than one answer to each element of work. The various elements of work that were identified were divided into three sub-groups: (i) preparations (ii) direct patient contact (iii) follow-up. The responses were used to formulate more specific questions in the second round to gain a more thorough understanding of the various elements of work involved in the monitoring of warfarin. In the second round, a list of all elements of work associated with INR monitoring identified in the first round by the whole panel was sent to the respondents prior to the new interview. The respondents were then asked to estimate the time required for executing the various elements of work and how frequently each occurred. In the third round, the respondents had the opportunity to revise their estimate of time based on their initial estimate and the group responses. The interviewer also totalled the time for the various elements of work and prompted the respondents as to whether this time appeared to be reasonable.

### INR tests taken in the patient's home (panel II)

The objective of panel II was to assess the resource consumption related to the INR monitoring episodes in patients with CAF during warfarin treatment when the INR test was taken in the patient's home.

A random sample of 106 district nurses from different PHC centres in 34 regions were invited to take part in the study. The sample of district nurses was stratified to ensure participation from all over Sweden. Up to three district nurses in each region were selected as primary respondents, and one additional for each PHC centre as stand-in if the primary respondent should be unable/unwilling to participate. The inclusion criterion was that each participating district nurse should manage no less than five CAF patients. However, this criterion had to be relaxed and redefined to "at least two patients", in order to be able to find a sufficient number of district nurses eligible to participate in the study. If both the primary and stand-in district nurses selected from a specific PHC centre failed to satisfy the inclusion criterion, the PHC centre was excluded and replaced by another PHC centre.

This Delphi-panel was performed in two rounds. The first round was accomplished by telephone interviews, and the second round by a postal questionnaire. The interviewer tried up to two times to contact the responder by telephone. If the respondent was not possible to reach, a reminder letter was sent out. If contact still failed, the stand-in nurse was asked to participate. Both the first and second round of the panels were carried out in December 2003.

The first round, a telephone interview, identified various elements of work associated with INR monitoring in the patient's home and canvassed the time required for each element of work. The frequency with which each element of work occurred and the distance to the patient's home was also investigated. The work was divided into sections, (i) preparations (ii) home visit and (iii) follow-up. In the second round, accomplished by a postal questionnaire, the question was reiterated and the respondents had the opportunity to revise their estimate of time based on their initial estimate and the group responses. After the completion of each section the respondent was asked if the total time was reasonable.

When estimating the resource consumption per element of work and responder, both the time required for carrying out each specific element of work and the frequency with which it occurred were taken into consideration.

### Statistical considerations

In the statistical analyses, the frequency of occurrence of each element of work was handled as quantitative, interval scale data. Standard descriptive statistical calculations were performed, such as arithmetical mean, minimum and maximum. The uncertainty in the estimates was illustrated by a 95% confidence interval (CI).

## Results

### INR tests taken at a PHC centre (panel I)

Of the 50 GPs invited to the study, 35 actually participated in the first round, 15 were primary respondents, 9 were first stand-ins and 11 were second stand-ins. In the second round one GP declined further participation and was replaced by a stand-in. In the third round 34 of the 35 GPs participated.

Ten of the 34 GPs collaborated with a registered nurse on a routine basis in the management of patients attending a PHC centre for INR monitoring. The remaining 24 GPs either collaborated with laboratory staff or an assistant nurse. A GP assisted by a nurse managed, on average, 40 patients with treatment on warfarin who visited the PHC centre for INR monitoring and a nurse managed, on average, 88 patients. GPs without nurse assistance managed, on average, 27 patients. Of all INR monitoring episodes, 11.6% of the blood samples were reported to be taken in the individual patient's home.

#### Management of patients

The daily routines for managing warfarin patients varied in and between the centres (Table 1). For GPs with an assistant nurse, the blood sample was always taken, analyzed and administrated by laboratory staff. For GPs without an assistant nurse, the blood sample in 74% of the cases was regularly managed by laboratory staff and in the remaining cases by nursing staff at the PCH centre. After the blood sample had been taken the patient normally left the PHC centre and the results of the INR test including change of dosage, if any, was communicated at a later time. The result of the test was always assessed by the GP and usually communicated to the patient by the GP. However, where a nurse routinely assisted a GP, the nurse shared this responsibility with the GP.

**Table 1:** Management of INR monitoring when tests are taken at PHC, (percentages).

**How INR tests are communicated between laboratory and nursing staff**	**GPs without nurse n = 25 (%)**	**GPs with nurse n = 10 (%)**	**Nurses n = 10 (%)**
Electronically	11 (44)	3 (30)	1 (10)
In writing ("Green Card")*	6 (24)	3 (30)	4 (40)
Verbally from nurse	3 (12)	3 (30)	3 (30)
Patient record (paper)	2 (8)	2 (20)	2 (20)

**Location of patient at time when receiving test result**	**GPs without nurse n = 25 (%)**	**GPs with nurse n = 10 (%)**	**Nurses n = 10 (%)**

At home	20 (80)	9 (90)	10 (100)
At PHC centre	10 (40)	4 (40)	3 (30)

**Test result communicated to patient**	**GPs without nurse n = 25 (%)**	**GPs with nurse n = 10 (%)**	**Nurses n = 10 (%)**

Telephone	21 (84)	8 (80)	9 (90)
In writing	7 (28)	5 (50)	3 (30)
Verbally	6 (24)	4 (40)	4 (40)
In writing "Green Card"	5 (20)	1 (10)	1 (10)

**Arranging new visit**	**GPs without nurse n = 25 (%)**	**GPs with nurse n = 10 (%)**	**Nurses n = 10 (%)**

In writing	9 (36)	1 (10)	2 (20)
Telephone patient	7 (28)	1 (10)	2 (20)
In writing "Green Card"	6 (24)	3 (30)	0 (0)
When prescription	4 (16)	2 (20)	4 (40)
When receiving test-results	5 (20)	2 (20)	1 (10)

*Green Card = a card held by the patient. N.B. Each respondent may score several items.

#### Time for INR monitoring

Total time for one INR monitoring episode for a *GP assisted by a nurse *(n = 10) was, on average, 10.1 minutes (CI 95% 5.4; 14.8), of which time for work preparation was 2.6 minutes, time for direct patient contact 3.1 minutes and time for follow-up 4.4 minutes. *Nurses *(n = 10) estimated total time for INR monitoring to 21.4 minutes (CI 95% 11.0; 31.8), on average, where work of preparation was 4.5 minutes, 3.9 minutes direct patient contact during the monitoring episode and 13.0 minutes for follow-up. Total average time for a *GP without nurse assistance *(n = 24) was 17.6 minutes (CI 95% 10.6; 24.6), of which time for preparation was 4.4 minutes, direct patient contact 6.1 minutes and time for follow-up 7.1 minutes. In table 2, frequency used and time required per element of work related to INR monitoring are given. Total average time for all participating GPs (n = 34) was 15.4 minutes (CI 95% 11.2; 19.6), of which time for work preparation was 3.9 minutes, time for direct patient contact was 5.2 minutes and time for follow-up was 6.3 minutes. Results of the estimates of time in the three rounds are shown in table 3.

**Table 2:** Frequency of activities and average time used per element of work when tests are taken at PHC centre (n = 34).

**Preparations**	**Average time used per element of work (minutes)**	**Range**
Take out patient's name	0.2	0.0–2.5
Take out patient's details	0.7	0.0–7.5
Read up on patient	1.0	0.0–15.0
Go through test results	0.6	0.0–5.0
Inform/discuss with nurse	0.3	0.0–5.0
Write lab. referral	0.2	0.0–3.0
Prepare patient information	0.3	0.0–10.0
Contact patient prior to checkup	0.7	0.0–13.5
**Total time for preparations**	3.9*	**0.0–18.4**

**Direct patient contact**	**Average time used per element of work (minutes)**	**Range**

Reception of patient prior to checkup	0.6	0.0–20.0
Collect patient from lab.	0.0	0.0–3.0
Personal discussion with patient	2.2	0.0–20.0
Inform patient of test results, new prescription and new appointment	1.3	0.0–15.0
Patient examination	1.1	0.0–30.0
**Total direct patient contact**	**5.2**	**0.0–17.2**

**Follow-up**	**Average time used per element of work (minutes)**	**Range**

Collect/take delivery of test results	0.3	0.0–5.0
Evaluate test results, new prescription	1.8	0.0–5.0
Complete and sign Waran form	1.0	0.0–5.0
Complete patient notes in computer	0.8	0.0–5.0
Complete patient notes in writing	0.2	0.0–3.0
Telephone patient	1.0	0.0–16.5
Write reply letter to patient	0.2	0.0–7.0
Write referral	0.2	0.0–3.0
Provide patient info to lab.	0.1	0.0–3.0
Provide patient info to nurse	0.6	0.0–5.0
**Total time for follow-up**	6.3*	**0.0–29.7**

***Total time used for one monitoring episode***	***15.4 minutes***	***1.4–49.8***

*the total is rounded off and thus does not correspond exactly with the sum of the individual items for follow-up

**Table 3:** Alteration in total time for INR monitoring between subsequent rounds, in minutes.

	**GPs without nurse, n = 25**	**GPs with nurse, n = 10**	**Nurses, n = 10**
First round	19.0	13.0	17.0
Second round	19.3	12.1	21.4
Third round	17.6*	10.1	-

*One GP dropped out (n = 24).

According to the GPs, the frequency with which patients failed to turn up for their INR monitoring visits was 11%, which the GPs estimated to consume 5.4 minutes (CI 95% 3.0; 7.8) of extra-work. Similarly, the nurses estimated the frequency of non-appearance to 17% and their assessment of the resulting extra-work was 4.9 minutes (CI 95% 2.0;7.8).

### INR tests taken in the patient's home (panel II)

Of the 106 district nurses, 50 nurses actually participated in the first round, 52 did not meet the criteria related to number of patient's managed, four nurses declined to participate and one nurse was impossible to reach. The results from the second round were based on 49 district nurses, due to one drop-out. Each district nurse was responsible, on average, for INR monitoring 4.9 CAF patients in their homes.

The estimated time required for INR monitoring averaged 90.5 minutes in the first round and 88.2 minutes in the second round. The total time for one INR monitoring episode was, on average, 88.2 minutes (CI 95% 76.5; 99.9), where time for work preparations was 23.9 minutes, direct patient contact during the home visit 23.4 minutes, and the time for follow-up after the home visit 40.8 minutes. In table 4, frequency used and time required per element of work related to INR monitoring is given.

**Table 4:** Frequency of activities and average time used per element of work when tests are taken in the patient's home (n = 49).

**Preparations**	**Average time used per element of work (minutes)**	**Range**
Take out patient's details	2.1	0.0–7.0
Read up on patient	2.3	0.0–10.0
Go through test results	1.9	0.0–10.0
Prepare patient information	2.1	0.0–18.0
Contact patient prior to checkup	2.0	0.0–10.0
Travel to patient	13.7	1.5–45.0
**Total time for preparations**	23.9*	**7.8–70.6**

**Home visit**	**Average time used per element of work (minutes)**	**Range**

Discussion with patient on warfarin treatment incl. info to patient	4.8	0.0–42.0
Discussion with patient on social implications	10.5	3.0–30.0
Take blood sample	4.8	1.0–20.0
Label and pack blood sample	3.2	0.1–15.0
**Total time for home visit**	23.4*	**7.1–76.5**

**Follow-up**	**Average time used per element of work (minutes)**	**Range**

Travel from patient	14.1	1.5–50.0
Delivery of blood sample to lab.	7.8	0.0–60.0
Receive test results and new prescription	4.6	0.0–20.0
Complete patient notes in computer	3.6	0.0–10.0
Complete written patient notes	0.5	0.0–5.0
Telephone patient regarding new prescription etc.	1.3	0.0–5.0
Write reply letter to patient	0.0	0.0–1.5
Travel to and from patient again	4.8	0.0–36.0
Personally inform patient of new prescription etc.	1.6	0.0–7.5
Divide up the Waran in dosett	2.5	0.0–15.0
**Total time for follow-up**	**40.8**	**8.0–146.1**

***Total time used for one monitoring episode***	***88.2 minutes***	***38.2–231.1***

*the total is rounded off and thus does not correspond exactly with the sum of the individual items for preparations, and direct patient contact.

Once a week, district nurses also made house calls to supply warfarin to patients' dose-dispending devices, and about half the patients also required the nurses' help to supply their medication. Some of these episodes demanded two home visits for each INR monitoring episode, one for the blood sample and one to supply medication. Each home visit required the district nurses to travel a mean distance of 13.7 km by car. About 8.2% of the home visits were in vain as patients were not at home, and the estimated mean extra time for such a fruitless visit was 44.6 minutes (CI 95% 33.1; 56.1).

## Discussion

In this study we assessed management and resource consumption associated with INR monitoring episodes in patients on warfarin treatment in PHC in Sweden. We found that such monitoring involves many elements of work, and demands large health care resources, especially when the INR test is taken in the patient's home. GPs, assistant nurses, laboratory personnel and district nurses take part in the monitoring most of the time, and as they have different responsibilities of coordination, the elements of work add time to carry out an INR monitoring episode. Another finding is that the time for follow-up after the test is taken is considerable, especially in home care.

PHC centres for the PHC panel (panel I) were solely recruited from the Stockholm County Council, where patients on warfarin are routinely managed in PHC. In many other places in Sweden such patients are partly cared for in anticoagulation clinics and partly in PHC. By choosing Stockholm County Council as the study area, confusion between different routines for management of the patients was thus avoided. In the panel where INR tests were taken in the patient's home (panel II) district nurses from all over Sweden took part. In this setting the distance to the caretaker is an important aspect, when assessing the resource use in the management of the patients, and a mixture of urban and rural areas was therefore felt to be necessary.

A limitation in our study is that it has only captured the time spent by GPs and nurses dedicated to INR monitoring visits, and district nurses. Further aspects of the work carried out by other types of personnel, e.g. laboratory staff and assistant nurses, have therefore not been included. The results obtained are therefore to be regarded as a conservative estimate of the resources required for carrying out INR monitoring episodes in PHC in Sweden.

The estimates of time decreased slightly in numerical value when the questions were reiterated in successive rounds. The respondents may have reflected over the actual time they spent on various aspects of INR monitoring episodes in between the rounds and therefore the final estimate is likely to be the most valid one. In some cases where GPs were nurse assisted there were discrepancies between what the GPs and the nurses had stated concerning different activities required when INR monitoring. It is notable, therefore, that nurses managed on average three times as many patients as doctors and it is therefore likely that they assisted more than one doctor in the PHC. It is also notable that the total time for a monitoring episode was considerable longer when both the GP and the nurse was involved.

In biomedical science, prospective, randomized, controlled studies performed under experimental conditions or trials are the generally accepted methodology for answering research questions within the health care sector. An alternative method to answer the research question at issue would have been to prospectively collect and measure resources required by observing the clinical daily routines in relation to INR monitoring of warfarin patients. An apparent weakness of using such a method is, however, that persons being observed in carrying out their work may intentionally or unintentionally change their behaviour, which may influence the result. However, it is doubtless that information based on attitudes and opinions is afflicted with uncertainty. Information from expert panels can, however, be gathered in a great number of ways, from informal discussions among a group of experts to standardised methods for gathering qualitative information. The Delphi method is an example of such a standardised method, and its methodology alleviates some of the problems experienced by other kinds of expert panel [14,15]. The process for carrying out the study preserves the integrity of the individual respondent without compromising the possibility to benefit from the knowledge of the other panel members. The reiterative procedure stimulates afterthought and gives the opportunity to modify responses, which is likely to increase the validity of the result of the study. We therefore believe the result of our study represents a good estimate of the management of and resources used by INR monitoring episodes in PHC in Sweden. Our results may therefore be used in further studies to explore the health care costs related to monitoring of warfarin in PHC, including unit costs of all individual costs items, and to address the economic impact of warfarin treatment.

## Conclusion

Monitoring of patients during treatment with warfarin in PHC in Sweden involves many elements of work, and demands large health care resources, especially when tests are taken in the patient's home.

## Competing interests

The study was supported by the Stockholm County Council and AstraZeneca Sverige AB. SA is a senior health economist at AstraZeneca Sverige AB.

## Authors' contributions

SA, IB, GHN and IK participated in the design and coordination of the study and drafted the manuscript. SA performed the statistical analysis. All authors read and approved the final manuscript.

## Pre-publication history

The pre-publication history for this paper can be accessed here:


